# Protection by the NO-Donor SNAP and BNP against Hypoxia/Reoxygenation in Rat Engineered Heart Tissue

**DOI:** 10.1371/journal.pone.0132186

**Published:** 2015-07-06

**Authors:** A. Görbe, A. Eder, Z. V. Varga, J. Pálóczi, A. Hansen, P. Ferdinandy, T. Eschenhagen

**Affiliations:** 1 Cardiovascular Research Group, Department of Biochemistry, University of Szeged, Szeged, Hungary; 2 Department of Experimental Pharmacology and Toxicology, Cardiovascular Research Center, DZHK (German Centre for Cardiovascular Research), partner site Hamburg/Kiel/Lübeck, Hamburg, Germany; 3 Department of Pharmacology and Pharmacotherapy, Semmelweis University, Budapest, Hungary; 4 Pharmahungary Group, Szeged, Hungary; National Institutes of Health, UNITED STATES

## Abstract

In vitro assays could replace animal experiments in drug screening and disease modeling, but have shortcomings in terms of functional readout. Force-generating engineered heart tissues (EHT) provide simple automated measurements of contractile function. Here we evaluated the response of EHTs to hypoxia/reoxygenation (H/R) and the effect of known cardiocytoprotective molecules. EHTs from neonatal rat heart cells were incubated for 24 h in EHT medium. Then they were subjected to 180 min hypoxia (93% N_2_, 7% CO_2_) and 120 min reoxygenation (40% O_2_, 53% N_2_, 7% CO_2_), change of medium and additional follow-up of 48 h. Time-matched controls (40% O_2_, 53% N_2_, 7% CO_2_) were run for comparison. The following conditions were applied during H/R: fresh EHT medium (positive control), the NO-donor S-nitroso-N-acetyl-D,L-penicillamine (SNAP, 10^-7^, 10^-6^, 10^-5^ M) or the guanylate cyclase activator brain type natriuretic peptide (BNP, 10^-9^, 10^-8^, 10^-7^ M). Frequency and force of contraction were repeatedly monitored over the entire experiment, pH, troponin I (cTnI), lactate dehydrogenase (LDH) and glucose concentrations measured in EHT medium. Beating activity of EHTs in 24 h-medium ceased during hypoxia, partially recovered during reoxygenation and reached time-control values during follow-up. H/R was accompanied by a small increase in LDH and non-significant increase in cTnI. In fresh medium, some EHTs continued beating during hypoxia and all EHTs recovered faster during reoxygenation. SNAP and BNP showed small but significant protective effects during reoxygenation. EHTs are applicable to test potential cardioprotective compounds in vitro, monitoring functional and biochemical endpoints, which otherwise could be only measured by using in vivo or ex vivo heart preparations. The sensitivity of the model needs improvement.

## Introduction

Ischemic heart disease is the leading cause of death in the industrialized world, therefore, development of cardioprotective therapies are of great importance. In vitro drug screening and disease modeling is a constantly growing research field, since the replacement/minimization of experimentally sacrificed animals in biomedical research and in the drug development process is a social and ethical necessity. 3-dimensional engineered heart tissue (EHT) shows typical phenotypic features of native heart tissue and is applicable to cardiovascular drug screening [1,2] and modeling of cardiovascular disease such as myocardial hypertrophy [3] and inherited cardiomyopathies [4]. However, there is limited information on the response of EHT to acute ischemia/reperfusion [5] and the involvement of cardioprotective signaling pathways that have been discovered in intact hearts.

Nitric oxide is a well-known cardioprotective molecule, which was extensively studied during last decades in different models of ischemia/reperfusion injury and cardioprotection [6]. Besides its vasodilator effect, direct cardiocytoprotection has been previously shown in case of simulated ischemia (SI) in 2D rat neonatal cardiac myocyte cultures with administration of the NO-donor S-Nitroso-N-acetyl-D,L-penicillamine (SNAP) [7]. Furthermore, administration of SNAP has been shown to mimic preconditioning protection in mouse hearts [8]. Concentration-dependent protection of exogenous NO against simulated ischemia/reperfusion induced injury was detected in mouse embryonic stem cell-derived cardiomyocytes as well [9]. The NO-mediated cytoprotection mainly acts via intracellular elevation of cyclic guanosine monophosphate (cGMP), which has a common PKG-dependent downstream signaling pathway with natriuretic peptides [7,10,11]. B-type natriuretic peptide (BNP), the main natriuretic peptide in ventricular myocardium, has been shown to limit infarct size in rat hearts and protects neonatal cardiac myocytes against simulated ischemia/reperfusion via activation of cGMP-PKG pathway [7,10].

Two-dimensional (2D) cardiac myocytes cultures are suitable for the exploration of intracellular mechanisms of cardiocytoprotective agents [12], but information about fundamental cardiac parameters such as beating rate, contractile force and contraction kinetics remains limited at best. Moreover 2D neonatal cardiac myocyte cultures are mixed with fibroblasts, of which the percentage and growing rate is difficult to control and unstable over time. Three-dimensional (3D) EHT could combine advantages of in vitro and in vivo/ex vivo experimental setups. The aim of the present study was to evaluate the suitability of EHT as an in vitro model of hypoxia/reoxygenation injury as well as a screening bed for cardioprotective interventions.

## Materials and Methods

### Cell isolation and EHT generation

The investigation conforms to the guide for the care and use of laboratory animals published by the NIH (Publication No. 85–23, revised 1985). Experimental procedures were reviewed and approved by Ethics Committee, University of Hamburg.

Neonatal rat cardiac myocytes (NRCM) were isolated from 1–3 day old neonates (Wistar and Lewis rats) and EHTs were generated as previously described [1,13]. In brief, rats were sacrificed by decapitation and hearts were digested with a combined enzymatic (DNAse/Trypsin) and mechanic treatment. EHTs were generated from the unpurified heart cell mix, using a reconstitution solution as follows (final concentration per ml): 4.1 x 106 cells, 3 U thrombin (Biopur BP 11-10-1104), 5 mg bovine fibrinogen (Sigma F4753). To yield isotonic conditions, 2xDMEM (20% horse serum, GIBCO 26050; 4% chick embryo extract; 2% penicillin/streptomycin, GIBCO 15140) was added matching the amount of fibrinogen and thrombin. Liquid agarose (2% in PBS; Invitrogen 15510–027), custom-made Teflon spacers and 24-well cell culture plates were used to prepare the casting molds. Silicone attachments (Silitec GmbH & Co. KG, Weiler-Simmerberg, Germany) with four pairs of silicone posts were placed onto the casting molds with each pair reaching into a mold. For each EHT 97 μl of the reconstitution mix was mixed separately with 3 μl thrombin and then pipetted into a mold. After 2 h of incubation (37°C, 40% O2, 7% CO2) the fibrinogen was polymerized, so that the EHTs could be transferred to a new, medium-filled (DMEM (Biochrom F0415) with: 10% horse serum, 2% chick embryo extract, 1% penicillin/streptomycin, insulin (10 μg/ml, Sigma I9278), aprotinin (33 μg/ml, Sigma A1153) cell culture dish. EHTs were fed every other day and maintained under cell culture conditions for up to four weeks. EHTs were kept at 40% oxygen throughout their development because systematic comparisons had shown higher force development at 40% than at standard 21% oxygen [14]. Experiments were done on EHTs in the period of day 15 to 22.

### Contractility measurement

To evaluate the contractile parameters of the EHTs, a setup for video-optical recordings was used. It consisted of a small cell incubator with controlled humidity, temperature (37°C) and gas composition (e.g. 40% O_2_, 7% CO_2_, 53% N2 for baseline/reoxygenation or 0% O_2_, 7% CO_2_, 93% N_2_ for hypoxia). This cell culture unit had a glass roof with a Basler CCD-camera (Type A 602f-2) mounted on a XYZ-device (IAI Corporation) above. A custom made software (Consulting Team Machine Vision, ctmv.de; Pforzheim, Germany) allowed to set camera positions for each EHT in a PC-controlled manner. Light-emitting diodes (LEDs) illuminate the EHTs during recordings, to which the light exposure was synchronized. The software detected the EHT at top and bottom end during recordings via a figure recognition mode. Contractile parameters (e.g. beats per minute, force) were calculated with an equation [15] based on the elastic modulus of the silicone (1.7 MPa), the geometry of the posts and the post deflection (delta value of post distance) during the contraction. Reports showing all calculated parameters were generated by the software automatically.

### Hypoxia/reoxygenation

**Fig 1:**
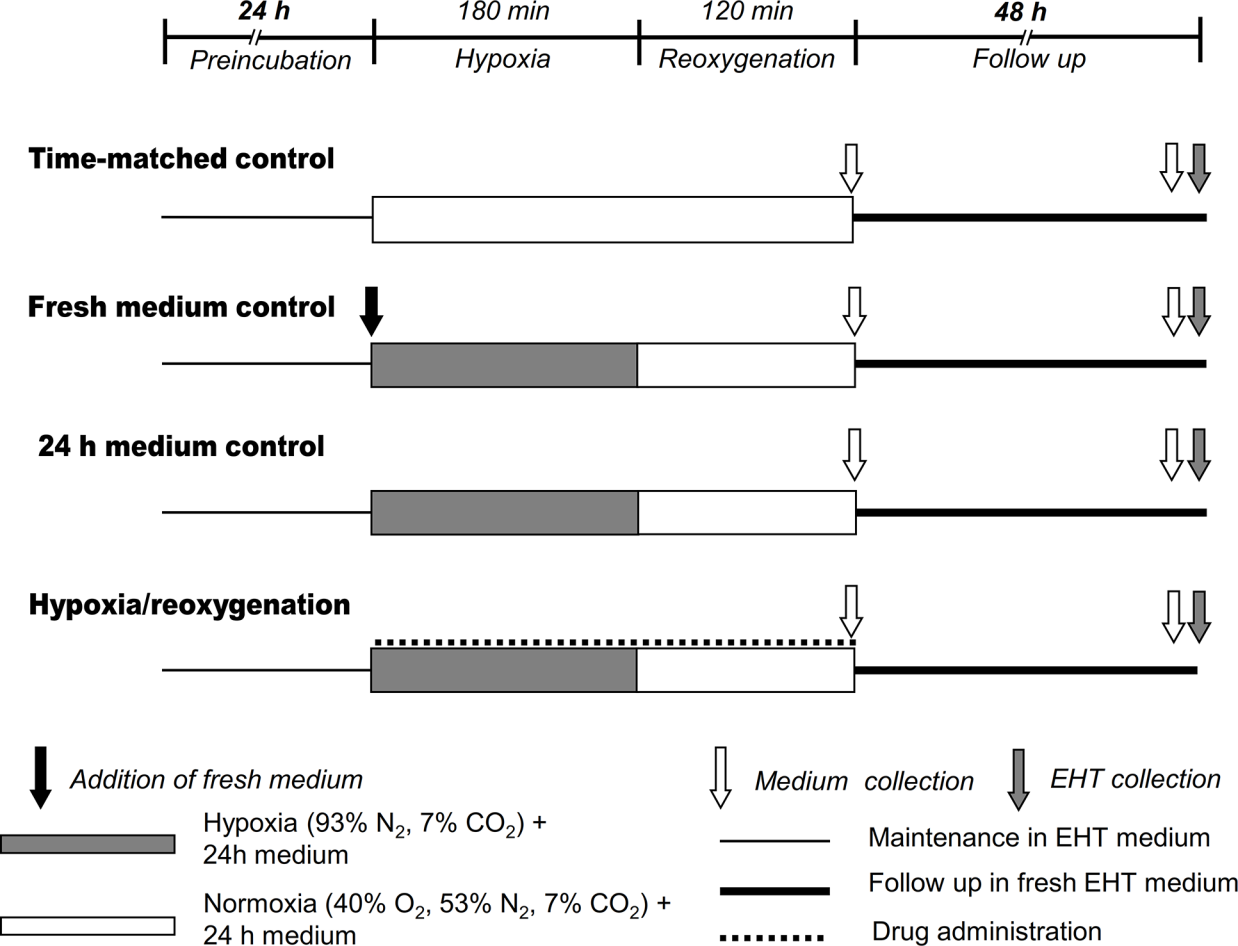
Study design and experimental groups.

To test EHT responses under ischemic stress, we used hypoxia/reoxygenation to simulate in vivo myocardial ischemia/reperfusion injury. For this purpose, the spontaneously contracting 15–22 days old EHTs were placed into fresh EHT medium 24 h before experiments. As a pilot experiment, 4 EHTs were subjected to hypoxia by culturing them in 93% N_2_ and 7% CO_2_. Based on their beating activity the 3 h hypoxic period was chosen for further experiments. EHTs were subjected to 180 min hypoxia (93% N_2_ and 7% CO_2_, EHTs were covered by 24 h conditioned medium) followed by 120 min of reoxygenation (40% O_2_ 53% N_2_ and 7% CO_2_, EHTs were covered by 24 h conditioned medium). Endpoints were recorded before hypoxia (baseline), at 11 time points during 180 min hypoxia (0, 15, 30, 45, 60, 80, 100, 120, 140, 160, 180 min) and at 8 time points during reperfusion (0, 20, 40, 60, 75, 90, 105, 120 min). At the end of experimental protocol the EHT medium was collected, and pH was determined. EHTs were placed into freshly prepared medium and were incubated further for 48 h. Additional 3 time points were recorded during the follow up period (overnight, 1 day, 2 days). In the end EHT medium was collected and pH was determined as previously (Fig 1).

### Experimental groups

Experimental groups are shown in Fig 1. To demonstrate the effect of simulated hypoxia/reoxygenation, EHTs subjected to hypoxia/reoxygenation (named 24 h medium control group; 24 h MC) was compared to the time-matched (normoxic) controls (TMC) that were kept in normoxia for 180+120 min. To demonstrate if the response of EHTs to ischemic stress was affected by known cardiocytoprotective drugs, S-Nitroso-N-acetyl-D,L-penicillamine (SNAP 10^−7^, 10^−6^, 10^−5^ M) (Sigma;St. Louis, MO) or brain type natriuretic peptide-32 (BNP, 10^−9^, 10^−8^, 10^−7^ M) (American Peptides; Sunnyvale, CA) were administered into the 24 h conditioned medium during the 180 min hypoxia and 120 min reoxygenation period.

### Cardiac troponin I, lactate dehydrogenase and glucose measurement in the supernatant

Medium of EHTs was collected at the end of the reoxygenation and follow up period. Cardiac troponin I (cTnI) concentrations were measured by a double-antibody sandwich enzyme-linked immunosorbent assay (ELISA) according to the manufacturer's protocol (Life Diagnostics, Inc., 2010-2-HSP). LDH release was measured with an LDH-P kit (Diagnosticum, Budapest, Hungary) with spectrophotometer. LDH release was expressed as U/ml. Glucose content of medium was measured by a hexokinase assay (Glucose HK MOD P, Roche, R11876899) and expressed in mmol/L. The pH of EHT medium was checked by pH test strips.

### Histological analysis

EHT samples were fixed in 10% neutral buffered formalin prior to paraffin embedding. 5 μm thick slices were made and stained with hematoxylin-eosin (HE) for the visualization of cellular structures. The central region of the longitudinally sectioned EHTs was visualized and examined with a light microscope (Nikon labophot-2) and images were acquired with Olympus DP71 colour camera using a 40× objective lens. An automatic quantification of nuclei and cellular parameters for estimating apoptosis were obtained from HE-stained sections using the CellProfiler 2.1.1 image analysis software (Broad Institute). Ten randomly chosen fields from the central region of each EHT were analyzed for nuclei diameter and area, whereas the area of parent cytoplasm of each nucleus was also captured and measured.

### Statistical analysis

Results are expressed as mean±SEM. The following statistical tests were applied: unpaired t-test and one way analysis of variance (ANOVA) followed by Dunett’s posthoc test; for multiple comparisons: multiple t-test and two way analysis of variance (ANOVA) followed by Dunett’s posthoc test. Differences were considered significant at p<0.05. Chi-squared test was used for the analysis of the beating map.

## Results

### Effect of hypoxia/reoxygenation

**Fig 2:**
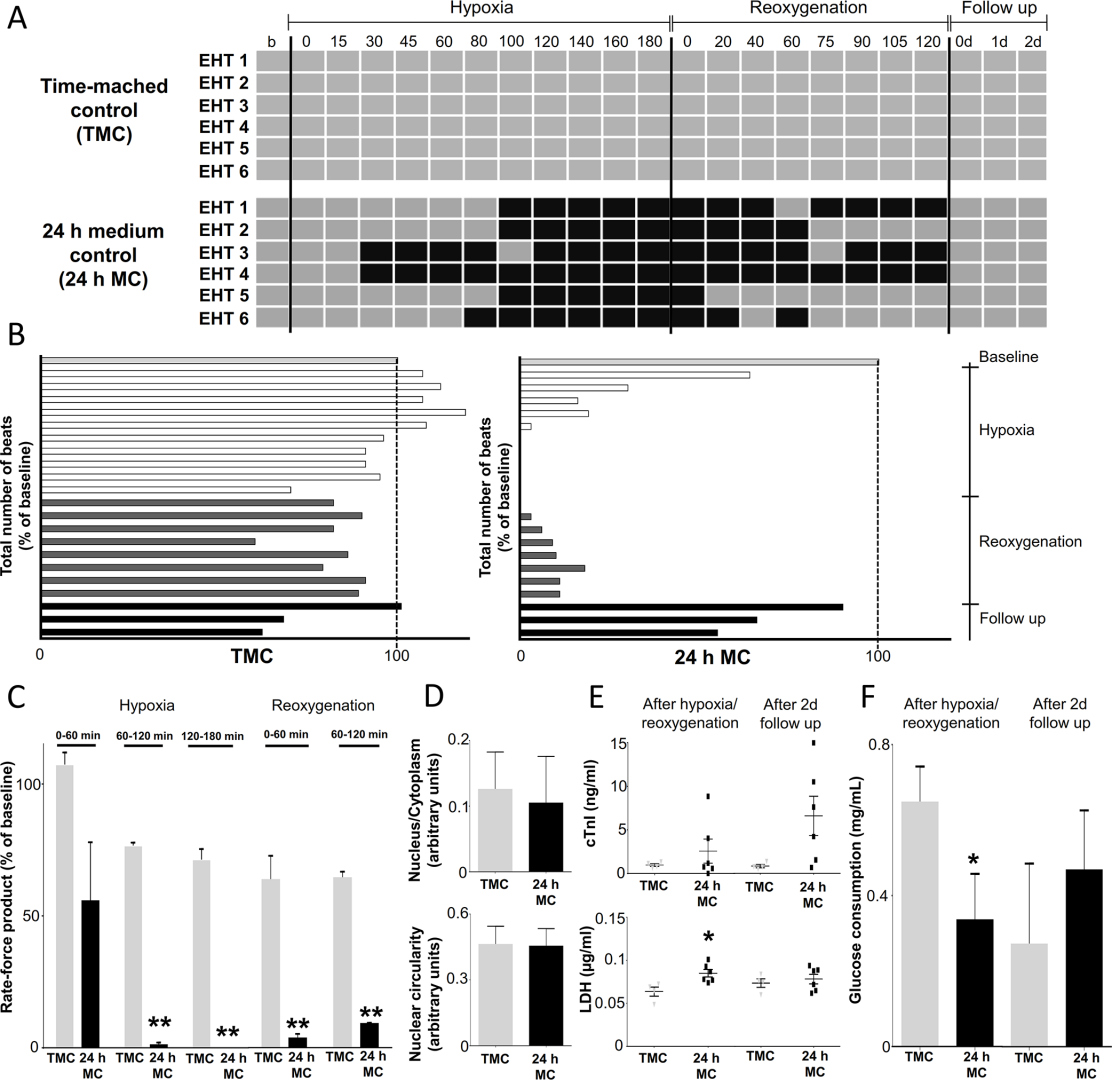
Comparison of EHT contractile behaviour in time-matched control (TMC) and 24 h medium control (24 h MC) groups (n = 6). (A) Beating pattern of EHTs was recorded during the entire experiment. Grey boxes indicate beating periods of EHTs, black boxes non-beating phases. (B) Total number of beats is expressed as the percentage of baseline. (C) Rate force product of EHTs. Data are expressed as mean ± SEM; **p<0.01 multiple t-test, n = 4–6. (D) Histological analysis of EHT sections (nucleus/cytoplasm ratio, nuclear circularity). (E-F) Biochemical markers of tissue necrosis (cardiac troponin I (cTnI) and lactate dehydrogenase (LDH) release after hypoxia or after 2 days follow up respectively) and glucose consumption are presented respectively. Data are expressed as mean ± SEM; *p<0.05; unpaired t-test, n = 4–6.

Spontaneously beating 15–22 days old rat EHTs, incubated for 24 h in EHT medium, were exposed to 180 min hypoxia and 120 min reoxygenation (24 h medium control; 24 h MC) or standard 40% oxygen (time-matched control; TMC). The beating map (Fig 2A) shows the contractile activity of EHTs at each recording time point (beating periods are expressed as grey boxes, non-beating periods are expressed as black boxes). TMC group were essentially stable during the whole experiment, while hypoxia induced a fast decrease in beating rate and complete cease after ~100 min. Three out of 6 EHTs recovered during reoxygenation and all of them during the follow up period.

The summary of all beats of each individual EHT (n = 6) at all recording time points showed a bell-shaped pattern after hypoxia in (Fig 2B) in the 24 h MC group. Time-matched controls showed a small decline in beating activity during the 5 h long recording period, probably due to metabolic changes. Hypoxia resulted in a pronounced decrease, while reoxygenation caused a fast re-initiation of beating rate, which did not reach TMC values during the reoxygenation period and remained under 20% of the baseline (S1 movie). Since rat EHTs have a negative force-frequency relationship, frequency multiplied by forces shows less biased results than frequency alone. The rate-force product (beat × force) was also significantly less in the 24 h MC group than in the TMC group at any stage of reoxygenation (Fig 2C). Follow-up did not show significant differences between the 24 h MC and TMC groups.

The decreasing nucleus/cytoplasm (N:C) ratio is a widely used cytologic marker for apoptosis. Circularity is another marker indicating the severity of apoptosis. N:C ratio and nuclear circularity did not change in 24 h MC group as compared to TMC group (Fig 2D).

The severity of the hypoxic injury of EHTs was monitored by troponin I and LDH release into the EHT medium during hypoxia/reoxygenation and follow up (Fig 2E). Glucose consumption was calculated by comparing the concentrations after the intervention with input concentrations (Fig 2F). The LDH concentration was significantly higher after hypoxia/reoxygenation in the 24 h MC group than in the TMC group, while troponin I showed only a tendency, which did not reach significance due to high variability. cTnI concentrations seemed also higher in the 24 h MC than in the TMC group after 48 h follow up, but values showed large variability and no statistical significance. Glucose consumption during hypoxia/reoxygenation was significantly lower than under control conditions, but did not differ during follow up. The pH of the medium was 7.5 after hypoxia/reoxygenation and at follow up in each group.

### Protective effect of fresh medium

**Fig 3:**
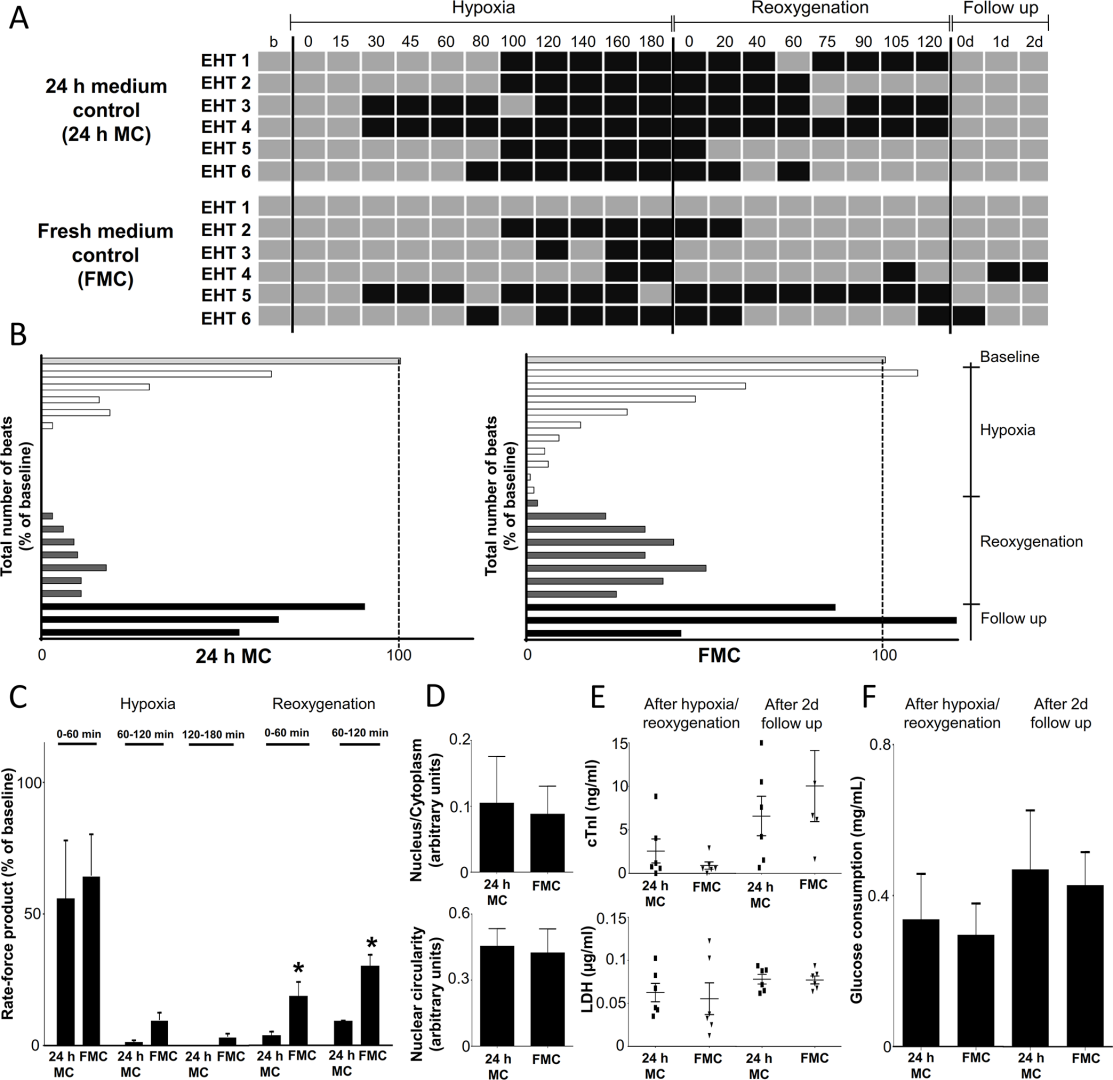
Comparison of EHT contractile behaviour in 24 h medium control (24 h MC) and fresh medium control (FMC) groups (n = 6). (A) Beating pattern of EHTs was recorded during the entire experiment. Grey boxes represent beating periods of EHTs whereas black boxes indicate non-beating phases. Beating pattern was analyzed by Chi square test showing significant differences among these groups (p<0.05). (B) Total number of beats is expressed as the percentage of baseline. (C) Rate force product of EHTs in 24 h MC and FMC groups during hypoxia and reoxygenation respectively. Data are expressed as mean ± SEM; *p<0.05; multiple t-test, n = 6. (D) Histological analysis of EHT sections (nucleus/cytoplasm ratio, nuclear circularity). (E-F) cTnI, LDH release and glucose consumption are shown respectively. Data are expressed as mean ± SEM; unpaired t-test, n = 6.

We noted in our pilot experiments (data not shown) that fresh medium had protective effects on EHTs and therefore used it as positive control. In fresh medium, EHTs continued to beat longer during hypoxia and reoxygenation or recovered quicker under reoxygenation (Chi-squared test, p<0.05 vs. 24 h MC; Fig 3A). Fresh medium-supplemented EHTs tended to beat further and restart earlier than the 24 h MC group, but the difference between groups did not reach statistical significance (Fig 3B).

The protective effect of fresh medium was seen under hypoxia, but was more pronounced during reoxygenation. Here, the rate-force product was several folds higher than in the 24 h MC group (Fig 3C). Nucleus/cytoplasm ratio and nuclear circularity, (Fig 3D, S1 Fig) or biochemical markers of tissue necrosis or glucose consumption did not differ between both groups (Fig 3E and 3F). The pH of the medium was 7.5 after hypoxia/reoxygenation and at follow up in each group.

### Cardiocytoprotective compounds

**Fig 4:**
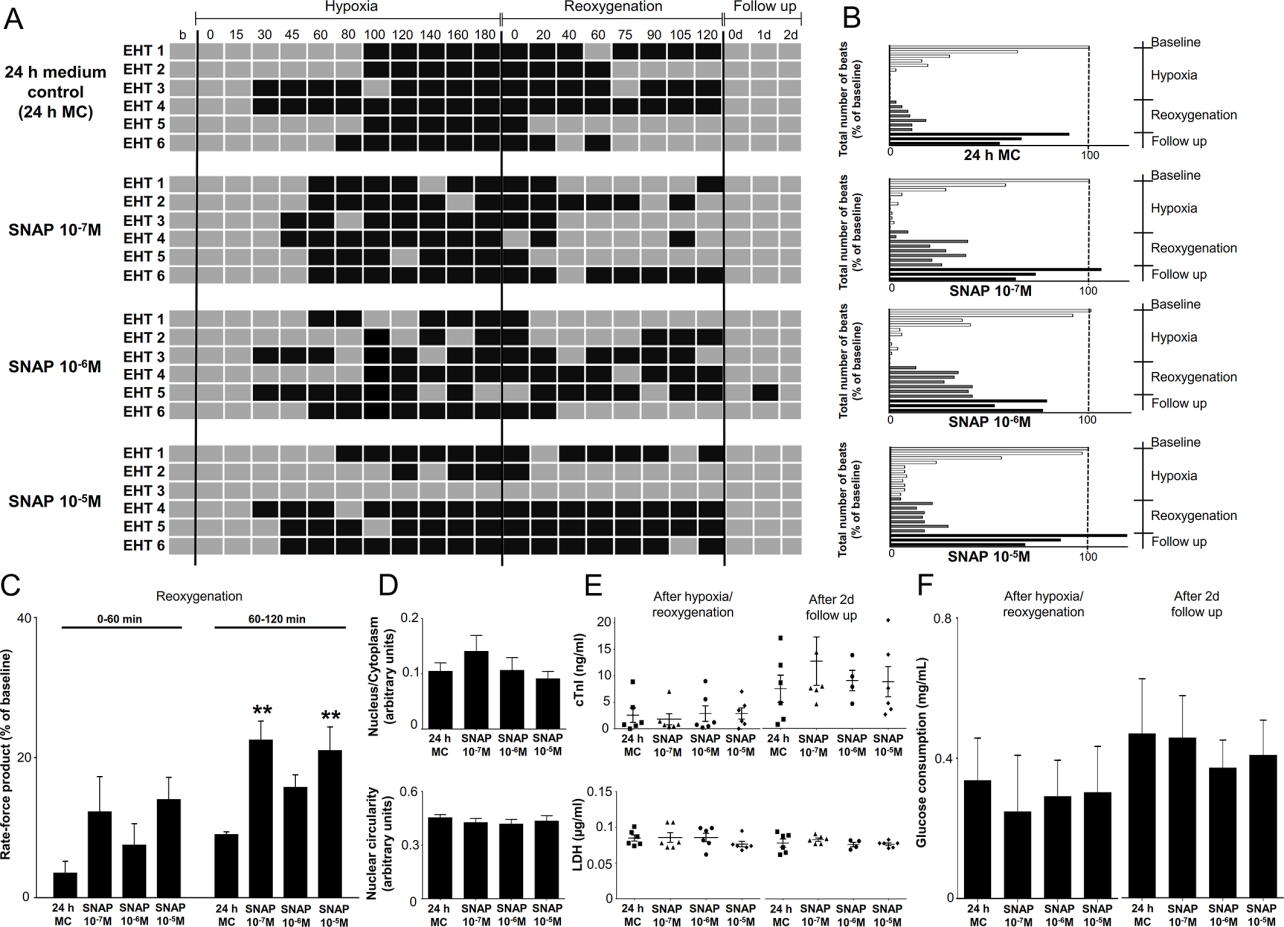
Comparison of EHT contractile behavior in 24 h medium control (24 h MC) and SNAP treated groups (n = 6). (A) Beating pattern of EHTs. Grey boxes represent beating periods of EHTs whereas black boxes indicate non-beating phases. (B) Total number of beats is expressed as the percentage of baseline. (C) Rate force product of EHTs during reoxygenation. (D) Histological parameters of EHT sections. (E-F) cTnI, LDH release and glucose consumption are shown respectively. Data are expressed as mean ± SEM; **p<0.001 one-way ANOVA, followed by Dunett’s post hoc test, n = 6.

The NO-donor SNAP or the B-type natriuretic peptide (BNP) were administered at 3 different concentrations to 24 h medium group before hypoxia/reoxygenation. Both had small, not clearly concentration-dependent effects on beating behavior of EHTs. None of the compounds caused significant protective effect during hypoxic phase (S2 and S3 Figs).In the presence of SNAP (10^−7^ M) more EHTs tended to beat during reoxygenation than in 24 h MC group (p = 0.06; Fig 4A). Beating activity (total number of beats; Fig 4B) was higher under SNAP 10^−7^ and 10^−6^ M, whereas the rate-force product during first stage of reoxygenation was not significantly changed, but during second stage of reoxygenation it was significantly higher under 10^−7^ and 10^−5^ M (Fig 4C). SNAP did not affect the N:C ratio (Fig 4D and S1 Fig) or biochemical markers (Fig 4E and 4F).

**Fig 5:**
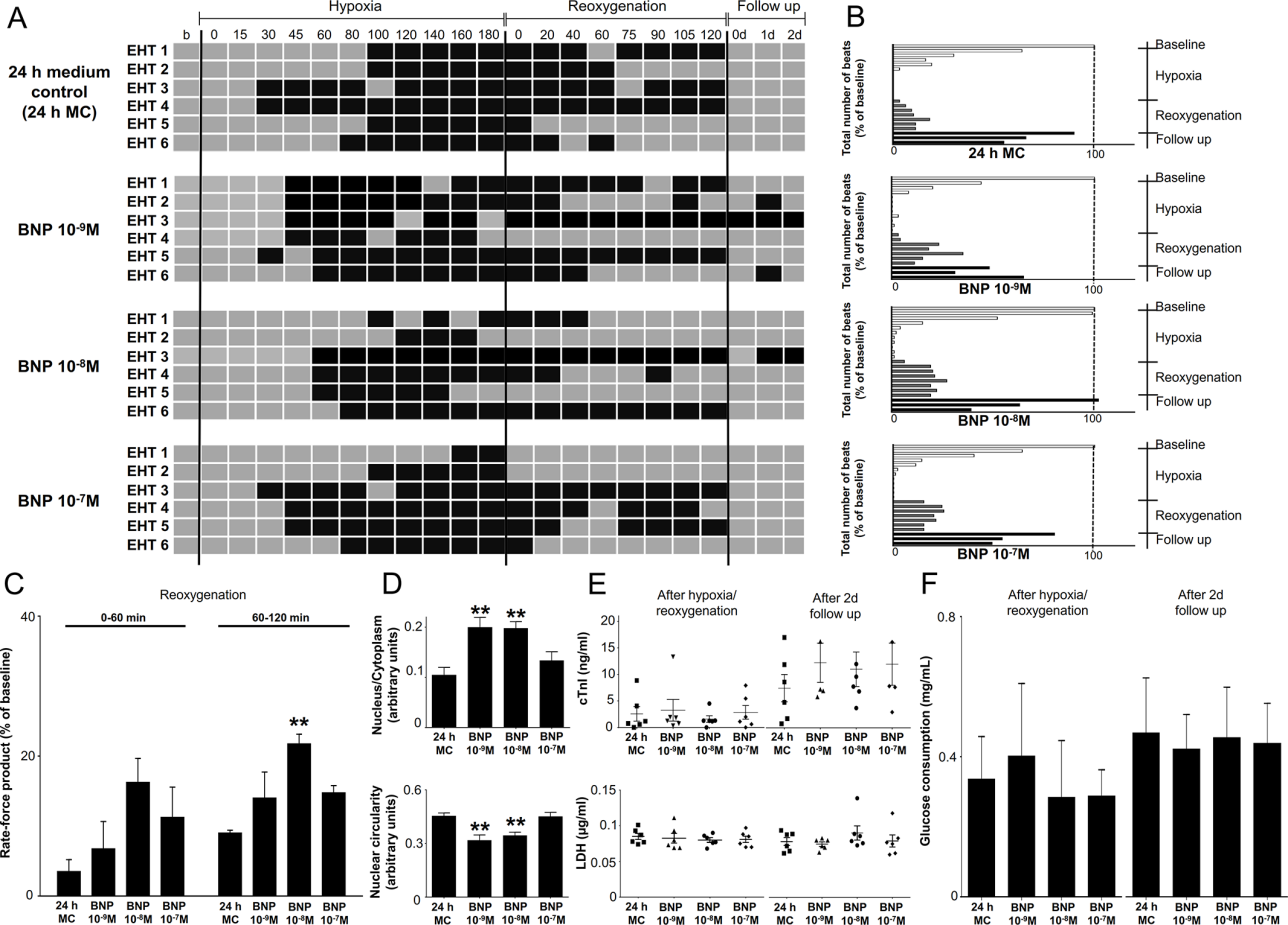
Comparison of EHT contractile behavior in 24 h medium control (24 h MC) and BNP treated groups (n = 6). (A) Beating pattern of EHTs. Grey boxes indicate beating periods of EHTs whereas black boxes show non-beating phases. (B) Total number of beats is expressed as the percentage of baseline. (C) Rate force product of EHTs during reoxygenation. (D) Histological parameters of EHT sections are visible. (E) cTnI and LDH release. (F) Glucose consumption. Data are expressed as mean ± SEM; **p<0.001 one-way ANOVA, followed by Dunett’s post hoc test, n = 6.

BNP did not affect the percentage of beating EHTs (Fig 5A). The sum of all beats (Fig 5B) and the rate-force product (Fig 5C) were all slightly higher than in the 24 h MC group with a maximal effect at BNP 10^−8^ M. The BNP (10^−9^ M, 10^−8^ M) treated group showed a significant increase the N:C ratio and nuclear circularity compared to 24 h MC (Fig 5D and S1 Fig). Biochemical markers and pH values did not differ among the groups (Fig 5E and 5F).

The diameter and length of EHTs were determined at each monitoring points (S4 and S5 Figs). Both, length and diameter of EHTs remained constant during the entire experiment.

### Time matched controls

The drop in rate-force product in the TMC group from the first to the second hour was unexpected and may be due to technical issues related to moving the 24-well-dish from the incubator to the set-up for video-optical measurements. None of the treatments had a systematic effect on any parameter (S6 Fig and S1–S6 Tables).

## Discussion

Here we report a novel in vitro model of hypoxia/reoxygenation injury that mimics some conditions of the ischemic heart in a 3-dimensional, tissue-like arrangement of cardiomyocytes (EHT). (i) EHTs showed a reproducible and fast response to hypoxia and reoxygenation, consisting of cessation of beating under hypoxia and slow recovery under reoxygenation. (ii) Hypoxia/reoxygenation in EHTs was associated with moderate LDH release. (iii) Significant functional recovery was observed after 48 h. (iv) Functional responses to hypoxia/reoxygenation were modified by medium conditions and agonists of the cGMP-PKG pathway that are known to protect against ischemia/reperfusion injury in other models.

Reliable in vitro test models for ischemia/reperfusion injury would be of great interest. However, available models are difficult to compare to the native heart in terms of histological features, contractile parameters, physiological and pharmacological responses. Therefore, there is an unmet need to develop cardiomyocyte-based test-beds for at least medium-throughput pharmacological screens. Previously, we have reported the responses of neonatal rat cardiomyocytes [7] and mouse embryonic stem cell-derived cardiomyocytes [9] to different concentrations of the NO-donor SNAP and B-type natriuretic peptide. Both test systems show good reproducibility and high-throughput but respond very differently to hypoxia/reoxygenation and drugs than the adult heart. For example, the adult heart is highly susceptible to ischemia/reperfusion injury. Upon global ischemia (less than 5 min) the heart shows no contractile activity and signs of stunning and hibernation [16]. In contrast, the widely used neonatal cardiomyocytes are relatively resistant to hypoxia/reoxygenation, therefore longer (minimum 150 min) hypoxia is required to induce cell death in this model [7,17]. In 2D cultures most of the cells are still alive after 150–240 min of simulated ischemia and continue to contract. They stop beating only after longer ischemic period (6–12 h). Embryonic stem cell-derived cardiomyocytes are also relatively resistant to hypoxia/reoxygenation, as we have reported previously [9].

Isolated adult cardiac myocytes show an intermediate phenotype. Qui et al showed that isolated rat cardiac myocytes have a reduced contractile function after 180 min ischemia [18] Furthermore, they showed this ischemic insult resulted in 70% cell death. Moreover, others have shown that isolated mouse ventricular myocytes are vulnerable for a 40 min ischemic insult and 50% of cells are apparently dead after 18 h reperfusion [19]. Isolated human cardiac myocytes are still contracting after isolation, too as well [20].

The data suggest that the degree of hypoxia-resistance reflects the developmental stage of the cells. Newborn rat survive up to 30 min in pure CO_2_ (ie, hypoxia and hypercapnia), whereas 10-day-old rats die after 5 min [21]. The fact that EHTs stopped beating quickly after the onset of hypoxia, needed several hours to fully recover after 180 min hypoxia and showed some increases in LDH indicates that this preparation has a higher maturity than 2D neonatal rat cardiac myocytes. On the other hand, they are still much less sensitive than adult cardiac myocytes. Glucose consumption rate, a measure of metabolic activity, correlated well with the amplitude of contraction in artificial heart tissue constructs [22]. EHTs showed a higher sensitivity to the hypoxic insult than neonatal rat heart cells cultured in 2D [7], indicating more aerobic glucose metabolism.

Besides the functional and biochemical parameters, signs of mild hypoxic injury, like morphologic parameters (ie, nuclear circularity, N:C ratio) were evaluated in this study. Morphologic hallmarks of apoptosis including chromatic margination, nuclear condensation and fragmentation, which are early events in cell death [23]. This process is then followed by the fragmentation of cell membrane and release of numerous intracellular substances (ie, LDH) [24]. However, the mixed cell population of EHTs was evaluated in this study, as the hypoxic response of full EHTs were in the focus. The morphological pattern of EHTs varies from the edge to the central region [25] and moderately ischemic central regions did not show early signs of apoptosis. Interestingly, we observed that simulated ischemia (combination of low pH-solution and hypoxia) stopped beating activity of EHT during the first 15 min without any recovery during reoxygenation (data not shown). The mechanisms of different hypoxia resistance likely a different dependency on oxidative mitochondrial metabolism, but more work is needed to answer this question.

Besides the differences in cell death caused by hypoxia/reoxygenation injury, there are notable differences in the response to various drug treatments. Both the NO- and the BNP-dependent cGMP-PKG pathways exerted remarkable cardioprotection in animal models of ischemia/reperfusion injury [10,26]. The cardioprotective importance of these pathways was also evidenced in neonatal cardiomyocytes [7], but only NO-dependent protection was detectable in mouse embryonic stem cell-derived cardiomyocytes [9]. In the present study, we describe moderate protection of EHTs against hypoxia/reoxygenation-induced stunning by NO and BNP. Both treatments induced apparent cardioprotection that was detected as a maintained contractile activity and inotropy. In the present study, SNAP significantly increased rate force product values at 10^−7^ M and 10^−5^ M, but not at 10^−6^ M during reoxygenation. The lack of significant protective effect of 10^−6^ M could be simply due to biological variations of the EHTs. However, the majority of studies failed to observe clear concentration-response relationships of NO donors including SNAP (see for reviews: [6,27]). This phenomenon may be due to the fact that local tissue NO concentrations largely depend on the ratio of NO and local superoxide production. The protection afforded by SNAP was more pronounced in comparison to BNP in the present study, which is in line with previous literature data obtained in conventional cellular models [28]. NO donors also attenuated the harmful effects of stunning, an effect comparable to that of late preconditioning [29,30]. In the present study, the protective effect of BNP showed a bell-shaped concentration-response curve with a maximum protection obtained at 10^−8^ M during reoxygenation. Bell-shaped concentration-response curves can be frequently observed with other cardiocytoprotective compounds, such as e.g. biglycan in neonatal rat cardiac myocytes [17] or SNAP in mice in vivo [31]. A similar observation was described by Burley and Baxter, showing that BNP at 10^−8^ M administered during reperfusion in isolated rat hearts is cardioprotective [10].

A notable limitation of the present study is that the degree of hypoxia/reoxygenation-induced injury as well as the protective effects of drugs was moderate. LDH, an indicator of cell death, only modestly increased after extended hypoxia/reoxygenation and contractile function fully recovered after 48 h. The magnitude of functional alterations (rate force product, time to first stop) did not correlate with necrosis and apoptosis markers, probably due to small differences between individual EHTs and the mild degree of hypoxic stimulus. Future work should be directed towards improved cellular maturation to increase the sensitivity of the assay to a level better comparable with adult hearts. Moreover, more harmful conditions should be found, so that the EHTs show a higher degree of injury and thereby give the opportunity to rescue function/biochemical parameters in a larger range.

Taken together we have shown for the first time that EHTs can be utilized as an experimental in vitro model for hypoxia/reoxygenation injury and cardioprotective drug treatment. Advantages compared to existing in vitro models are the possibility to monitor several parameters of contractile function under highly controlled conditions and, perspectively, to switch to a human, pluripotent stem cell-derived EHT model.

## Supporting Information

S1 Fig Representative micrographs of hematoxylin/eosin stained engineered heart tissue sections. **Arrowheads indicate apoptotic cells.** Scale bar: 20 μm.
(TIF)

S2 Fig Comparison of EHT rate force product in 24 h medium control (24 h MC) and SNAP treated groups (n = 6) during hypoxia. Data are expressed as mean ± SEM; **p<0.001 one-way ANOVA, followed by Dunett’s post hoc test, n = 6.
(TIF)

S3 Fig Comparison of EHT rate force product in 24 h medium control (24 h MC) and BNP treated groups (n = 6) during hypoxia. Data are expressed as mean ± SEM; **p<0.001 one-way ANOVA, followed by Dunett’s post hoc test, n = 6.
(TIF)

S4 Fig Diameter of EHTs expressed as percentage of baseline. (TIF)

S5 Fig Length of EHTs, expressed as percentage of baseline. (TIF)

S6 Fig Comparison of EHT contractile behavior in time-matched control groups (n = 4). Total number of beats is expressed as the percentage of baseline.
(TIF)

S1 Movie Contraction of EHTs (baseline, hypoxia, reoxygenation). (AVI)

S1 Table Rate-force product of time-matched controls during 3 h normoxia (suitable for the hypoxic period). Mean values are expressed in beats per min×mN.
(PDF)

S2 Table Rate force product of time-matched controls during 2 h normoxia (suitable for the reoxygenation). Mean values are expressed in beats/min× mN.
(PDF)

S3 Table cTnI release of time-matched controls. Mean values are expressed in ng/mL.
(PDF)

S4 Table LDH release of time-matched controls. Mean values are expressed in μg/mL.
(PDF)

S5 Table Glucose consumption of time-matched controls. Mean values are expressed in mg/mL.
(PDF)

S6 Table Histological analysis of time-matched controls. Mean values are expressed in arbitrary units.
(PDF)
