# Supplementary figures and images for: Protection by the NO-Donor SNAP and BNP against Hypoxia/Reoxygenation in Rat Engineered Heart Tissue
Source: PLoS One. 2015 Jul 6;10(7):e0132186. doi: 10.1371/journal.pone.0132186 (PMC4492769; doi:10.1371/journal.pone.0132186)

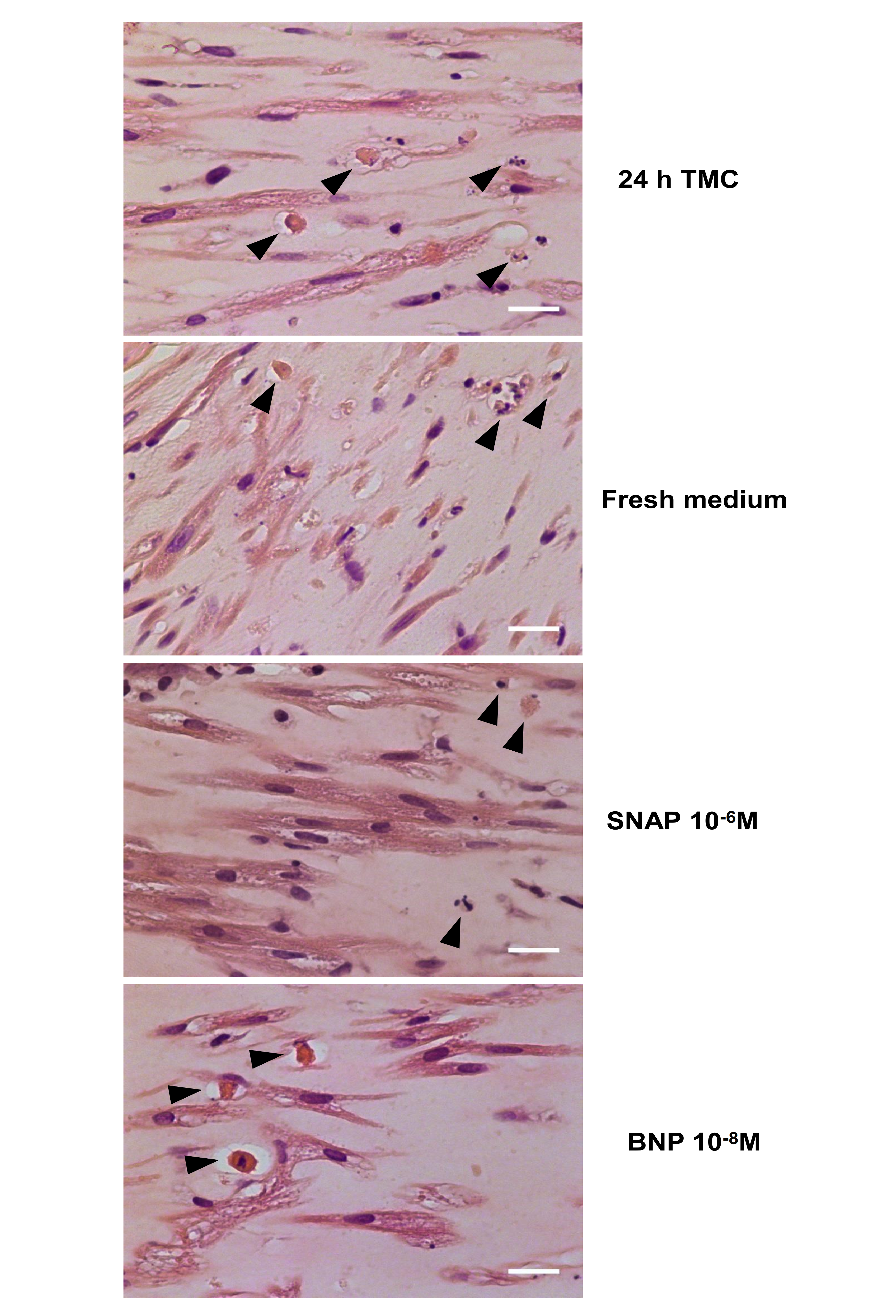

Supplement: S1 Fig — Arrowheads indicate apoptotic cells. Scale bar: 20 μm. (TIF) [file pone.0132186.s001.tif]

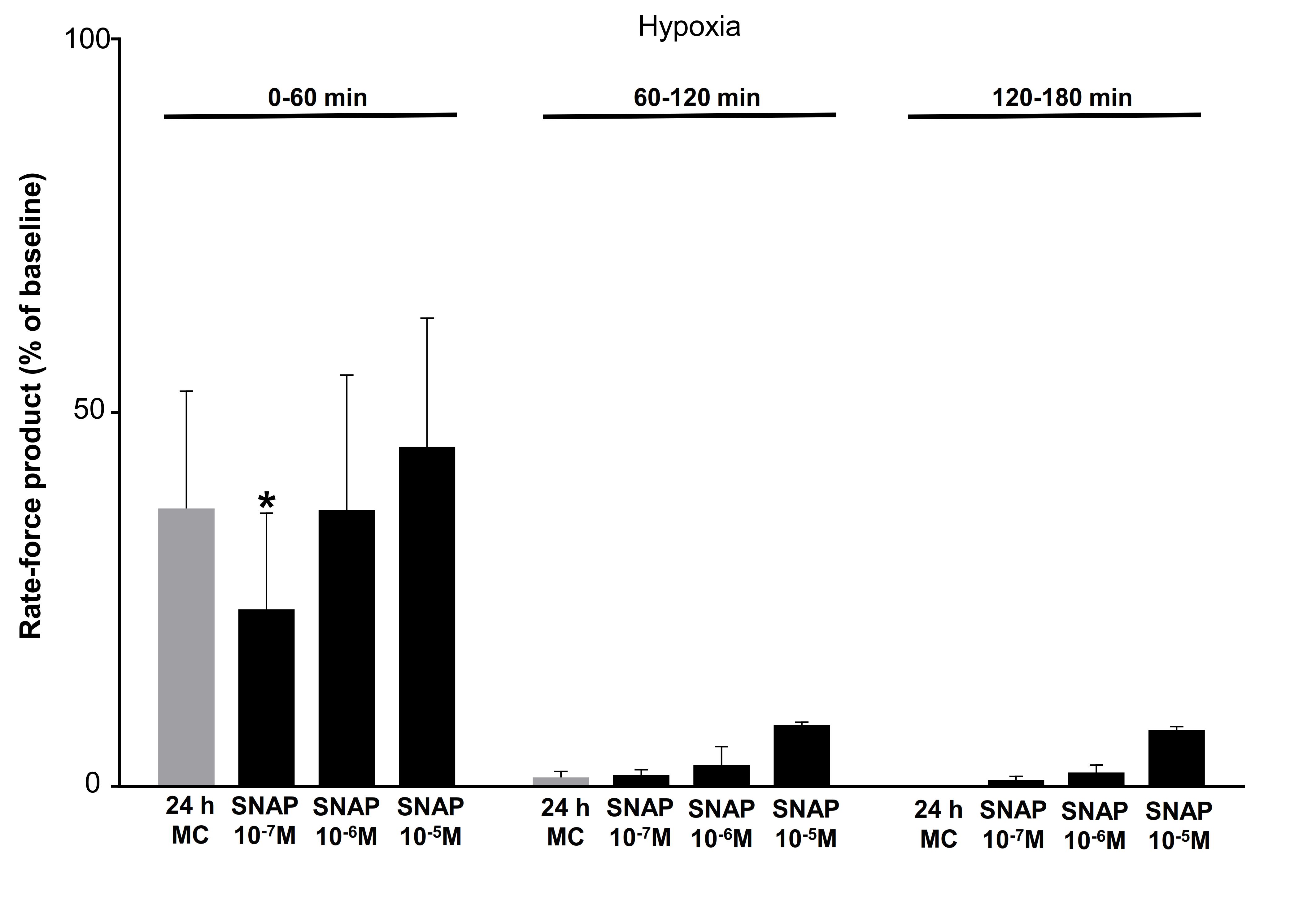

Supplement: S2 Fig — Data are expressed as mean ± SEM; **p<0.001 one-way ANOVA, followed by Dunett’s post hoc test, n = 6. (TIF) [file pone.0132186.s002.tif]

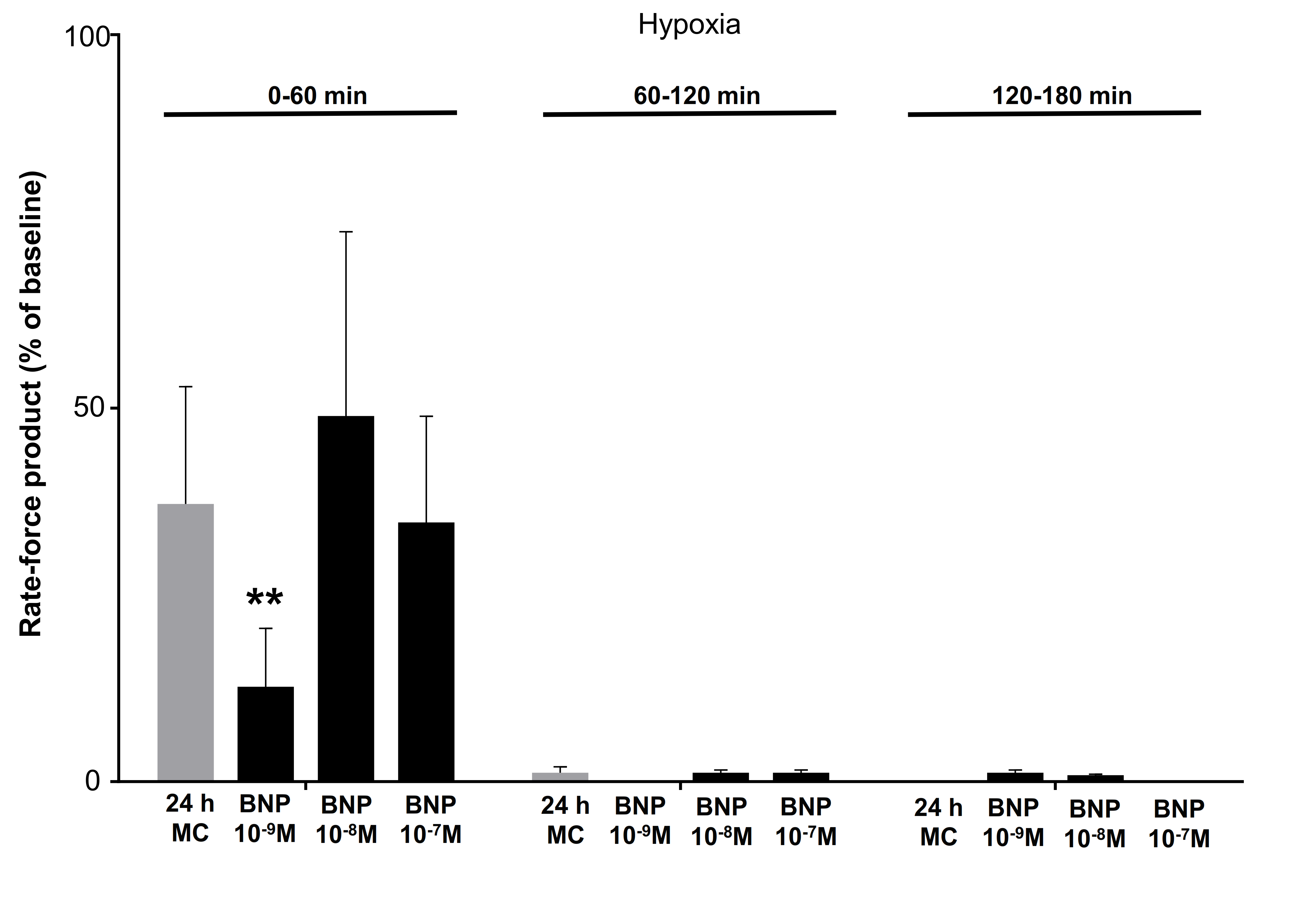

Supplement: S3 Fig — Data are expressed as mean ± SEM; **p<0.001 one-way ANOVA, followed by Dunett’s post hoc test, n = 6. (TIF) [file pone.0132186.s003.tif]

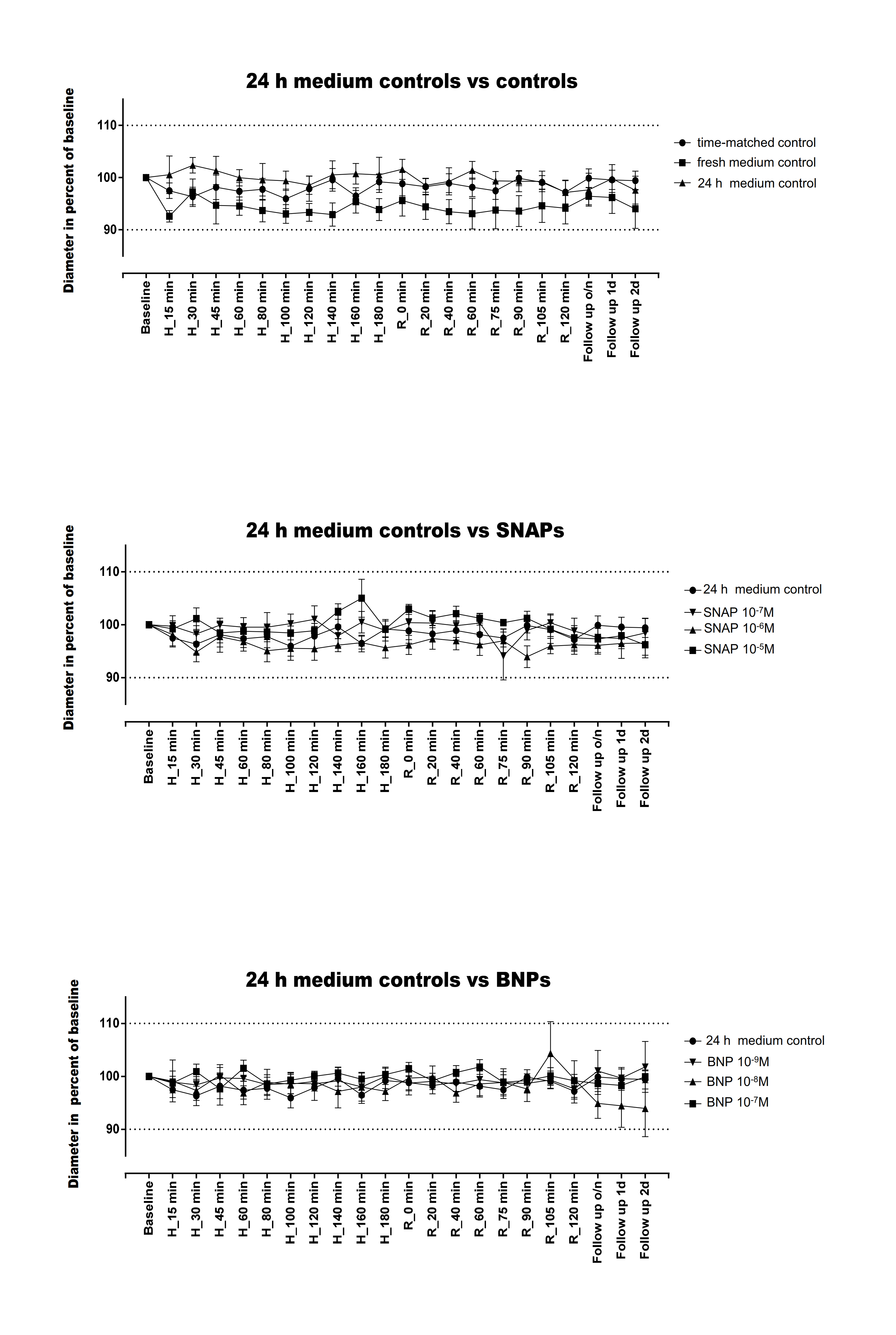

Supplement: S4 Fig — (TIF) [file pone.0132186.s004.tif]

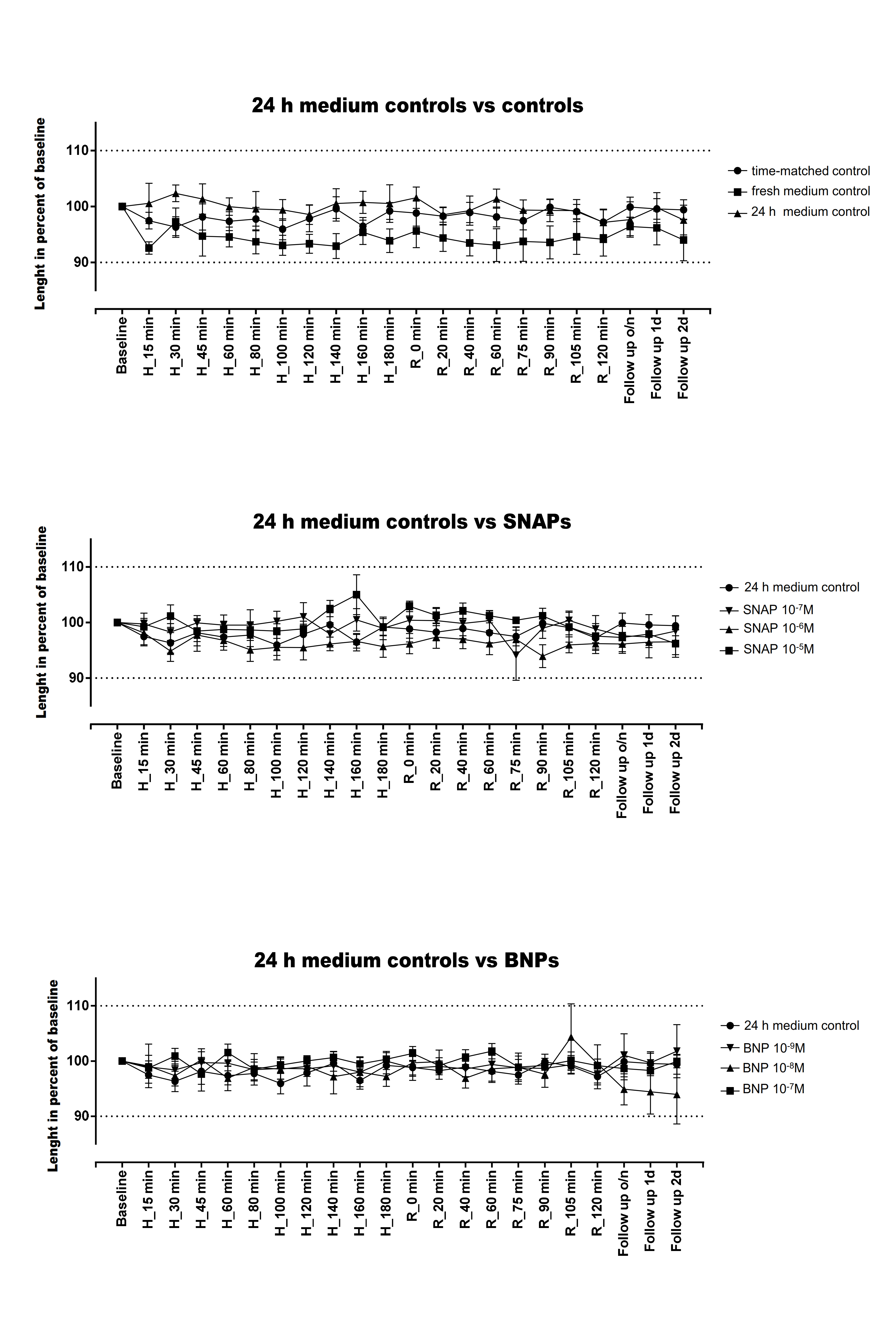

Supplement: S5 Fig — (TIF) [file pone.0132186.s005.tif]

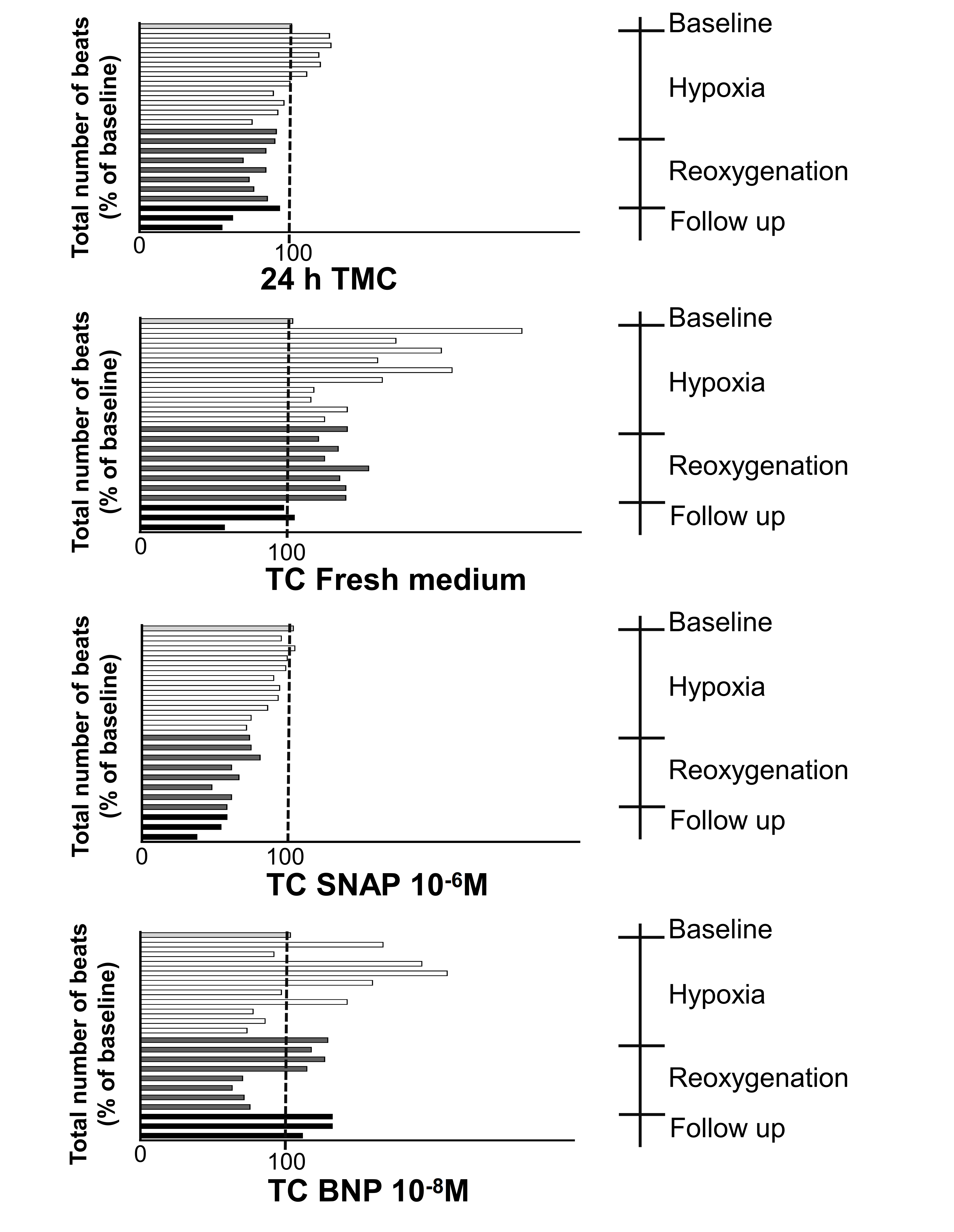

Supplement: S6 Fig — Total number of beats is expressed as the percentage of baseline. (TIF) [file pone.0132186.s006.tif]
