# Supplementary material for: Protection by the NO-Donor SNAP and BNP against Hypoxia/Reoxygenation in Rat Engineered Heart Tissue
Source: PLoS One. 2015 Jul 6;10(7):e0132186. doi: 10.1371/journal.pone.0132186 (PMC4492769; doi:10.1371/journal.pone.0132186)
Supplement: S1 Table — Mean values are expressed in beats per min×mN. (PDF) [file pone.0132186.s008.pdf]

Table 1. Rate-force product of time-matched controls during 3 h normoxia (suitable for hypoxic period). Mean values are expressed in beats/min× mN

	0-60 min		60-120 min		120-180 min	
Group	Mean±SEM	p value	Mean±SEM	p value	Mean±SEM	p value
24 h MC	99.44±5.26		93.25±5.75		95.33±9.30	
FMC	142.21±10.54	0.0093	95.01±18.35	0.9035	78.33±1.45	0.1717
SNAP (10^{-6}M)	94.21±6.04	0.7158	70.01±3.00	0.1335	62.33±2.40	0.0195
BNP (10^{-8}M)	143.40±15.51	0.0078	113.66±2.96	0.181	66.66±12.73	0.0352
