# Supplementary material for: Protection by the NO-Donor SNAP and BNP against Hypoxia/Reoxygenation in Rat Engineered Heart Tissue
Source: PLoS One. 2015 Jul 6;10(7):e0132186. doi: 10.1371/journal.pone.0132186 (PMC4492769; doi:10.1371/journal.pone.0132186)
Supplement: S2 Table — Mean values are expressed in beats/min× mN. (PDF) [file pone.0132186.s009.pdf]

Table 2. Rate force product of time-matched controls during 2 h normoxia (suitable for reoxygenation). Mean values are expressed in beats/min× mN

	0-60 min		60-120 min	
Group	Mean±SEM	p value	Mean±SEM	p value
24 h MC	90.59±14.48		120.33±22.31	
FMC	77.50±8.18	0.3253	78.50±3.27	0.035
SNAP (10^{-6}M)	63.01±4.22	0.0479	50.01±6.39	0.0018
BNP (10^{-8}M)	83.01±4.35	0.5555	73.51±8.38	0.0208
