# Supplementary material for: Protection by the NO-Donor SNAP and BNP against Hypoxia/Reoxygenation in Rat Engineered Heart Tissue
Source: PLoS One. 2015 Jul 6;10(7):e0132186. doi: 10.1371/journal.pone.0132186 (PMC4492769; doi:10.1371/journal.pone.0132186)
Supplement: S3 Table — Mean values are expressed in ng/mL. (PDF) [file pone.0132186.s010.pdf]

Table 3. Cardiac troponin I release of time-matched controls. Mean values are expressed in ng/mL.

	After hypoxia/reoxygenation		After 2 d follow up	
Group	Mean±SEM	p value	Mean±SEM	p value
24 h MC	0.96±0.16		0.94±0.21	
FMC	0.63±0.02	0.7818	8.33±6.78	0.2575
SNAP (10^{-6}M)	3.22±2.01	0.1078	4.46±1.93	0.58
BNP (10^{-8}M)	1.55±0.34	0.6903	9.33±5.41	0.1716
