# Supplementary material for: Protection by the NO-Donor SNAP and BNP against Hypoxia/Reoxygenation in Rat Engineered Heart Tissue
Source: PLoS One. 2015 Jul 6;10(7):e0132186. doi: 10.1371/journal.pone.0132186 (PMC4492769; doi:10.1371/journal.pone.0132186)
Supplement: S4 Table — Mean values are expressed in μg/mL. (PDF) [file pone.0132186.s011.pdf]

Table 4. LDH release of time-matched controls. Mean values are expressed in μg/mL.

	After hypoxia/reoxygenation		After 2 d follow up	
Group	Mean±SEM	p value	Mean±SEM	p value
24 h MC	0.063±0.005		0.073±0.004	
FMC	0.061±0.001	0.6141	0.067±0.005	0.4865
SNAP (10^{-6}M)	0.069±0.002	0.2782	0.075±0.004	0.8163
BNP (10^{-8}M)	0,067±0.003	0.4917	0.091±0.008	0.0694
