# Supplementary material for: Protection by the NO-Donor SNAP and BNP against Hypoxia/Reoxygenation in Rat Engineered Heart Tissue
Source: PLoS One. 2015 Jul 6;10(7):e0132186. doi: 10.1371/journal.pone.0132186 (PMC4492769; doi:10.1371/journal.pone.0132186)
Supplement: S5 Table — Mean values are expressed in mg/mL. (PDF) [file pone.0132186.s012.pdf]

Table 5. Glucose consumption of time-matched controls. Mean values are expressed in mg/mL.

	After hypoxia/reoxygenation		After 2 d follow up	
Group	Mean±SEM	p value	Mean±SEM	p value
24 h MC	0.648±0.046		0.273±0.106	
FMC	0.640±0.015	0.9211	0.446±0.013	0.159
SNAP (10^{-6}M)	0.612±0.031	0.6702	0.204±0.111	0.592
BNP (10^{-8}M)	0.464±0.104	0.0496	0.373±0.081	0.4014
