# Supplementary material for: Protection by the NO-Donor SNAP and BNP against Hypoxia/Reoxygenation in Rat Engineered Heart Tissue
Source: PLoS One. 2015 Jul 6;10(7):e0132186. doi: 10.1371/journal.pone.0132186 (PMC4492769; doi:10.1371/journal.pone.0132186)
Supplement: S6 Table — Mean values are expressed in arbitrary units. (PDF) [file pone.0132186.s013.pdf]

Table 6. Histological analysis of time-matched controls. Mean values are expressed in arbitrary units.

	Nucleus/Cytoplasm ratio		Nuclear circularity	
Group	Mean±SEM	p value	Mean±SEM	p value
24 h MC	0.125±0.013		0.461±0.019	
FMC	0.143±0.012	0.2423	0.433±0.010	0.2196
SNAP (10^{-6}M)	0.112±0.014	0.5412	0.429±0.017	0.1528
BNP (10^{-8}M)	0.146±0.013	0.3236	0.427±0.015	0.1721
